# Origin, Genetic Diversity, and Population Structure of Rabbits (*Oryctolagus cuniculus*) in Kenya

**DOI:** 10.1155/2019/7056940

**Published:** 2019-11-06

**Authors:** Sharon Auma Owuor, Edward George Mamati, Remmy Wekesa Kasili

**Affiliations:** ^1^Institute for Biotechnology Research, Jomo Kenyatta University of Agriculture and Technology, Juja, Kenya; ^2^Department of Horticulture and Food Security, Jomo Kenyatta University of Agriculture and Technology, Juja, Kenya

## Abstract

To evaluate the origin, genetic diversity, and population structure of domesticated rabbits in Kenya, a 263-base pair region of mtDNA D-loop region of 111 rabbits sampled from Kakamega, Vihiga, and Bungoma counties in the western region, Laikipia and Nyandarua counties in the central region, and Kitui, Machakos, and Makueni in the eastern region of the country were analyzed. The average haplotype (0.40702) and nucleotide (0.01494) diversities observed were low, indicating low genetic diversity of domesticated rabbits in Kenya. This study resolved 5 unique haplotypes in the mtDNA D-loop region. A population genetic structure distinguishing Europe grouping and domesticated rabbits in Kenya was obtained on incorporating 32 known haplotypes. Domesticated rabbits in Kenya clustered together with rabbits from other geographic regions, suggesting common origin. The results suggested that the Kenyan domesticated rabbits may have originated from Europe. Integration of exotic breeds into breeding programmes could have contributed to the low genetic diversity. These results provide useful information for breeding and conservation decisions by the relevant stakeholders in the agriculture industry in Kenya.

## 1. Introduction

Domesticated rabbits are descendants of the European rabbit, *Oryctolagus cuniculus*, which exists in both wild and domestic forms [1, 2]. The Iberian Peninsula is recorded to be the origin of domesticated rabbits, and two subspecies, *Oryctolagus cuniculus cuniculus* and *Oryctolagus cuniculus algirus*, coexist, with the former occurring in the north eastern part and the latter in the south west [3, 4]. Another possible center of domestication is the South of France, where human-mediated efforts such as migration are said to have introduced rabbits to this locality [1].

Rabbit rearing is a recent practice, and it is reported to have been followed by breeding which began in two localities, the Iberian Peninsula in first century BC and the ancient French monastery in Rome in the last 1,500 years [5]. Rabbit keeping in Kenya commenced in the 19^th^ Century when missionaries came to Kenya [6]. Collaboration between the Kenyan and German governments through the German International Development Agency, GIZ, saw the activity that was previously regarded as a hobby among teenage boys gain prominence, leading to promotion of rabbit farming through two major ways: 4K Clubs and establishment of breeding stations such as Rabbit Breeders Association of Kenya (RABAK).

Domestication is reported to have had an impact on genetic diversity as is evident in the immense phenotypic diversity present in domesticated rabbit. Phenotypic diversity is an evidence of the underlying genetic diversity [1]. Diversity studies are key for animal genetic resources mainly because they inform breeding programmes, enabling farmers and other relevant stakeholders to make informed decisions on which breeds are desirable for various traits such as high reproduction, disease resistance, and good meat production among other benefits. In the end, this contributes to food and livelihood security [7].

Application of molecular markers in diversity studies has been employed in a variety of animal species such as cows, goats, and sheep [8]. The commonly used molecular markers include microsatellites, amplified fragment length polymorphisms (AFLPs), random amplified length polymorphisms (RAPDS), and mtDNA (mitochondrial DNA).

Three key features that make mtDNA as an attractive marker for studying origin and diversity include high mutation rates, maternal inheritance, and availability in large quantities in the cell, facilitating easy isolation [9]. The high mutation rates enable accountability for the variation that a species undergoes over time, and maternal inheritance enables tracing all the animals to their ancestor(s). This facilitates diversity studies in animal species and has been applied in the current study of the origin of domesticated rabbits in Kenya.

Rangoju et al. [10] used RAPD markers to assess the genetic variability among three rabbit breeds: Soviet Chinchilla, White Giant, and Grey Giant. They reported no significant genetic variability among and within these three rabbit breeds. Surridge et al. [11] used microsatellites to investigate the genetic structure of European wild rabbits in East Anglia and reported that though they incorporated both ancient and recent populations, there was no evidence of loss in genetic diversity because of founder effects from population expansion. Alves et al. [1] used microsatellite markers to study the various patterns of genetic structure in domestic rabbits and quantify the diversity lost due to domestication and breed formation. This study reported what they termed as “directional loss in genetic diversity” from the wild rabbits in the Iberian Peninsula, heterozygosity (He) 0.825 to the domestic rabbits in France heterozygosity (He) 0.581. In another study, [1, 12] utilized microsatellites and mtDNA cytochrome b, respectively, in the study of genetic diversity of the rabbit. The former reported that wild rabbits from France were grouped together with wild rabbits from the Iberian Peninsula, whereas [12] revealed that the rabbits under the study belonged to lineage A, except a single rabbit that belonged to lineage G. Lineage A is the major lineage among wild rabbit populations, while lineage G is rarely reported in the studies on origin of rabbits.

Restriction fragment length polymorphism (RFLP) analysis of the mtDNA molecule by [13–15] revealed two maternal lineages, A and B, in the European rabbit. All domesticated rabbits are reported to belong to lineage B. Similarly, analyses based on immunoglobulin genes revealed a clear structuring into two mitochondrial clades, A and B [16]. Long et al. [2] determined diversity and origin of 104 rabbits from 20 rabbit by evaluating a 700 bp fragment of the mtDNA control region. The breeds comprised of three Chinese domestic breeds, twelve introduced breeds, and five derived breeds Their study obtained four new haplotypes that had previously not been reported and reported that the Chinese rabbits originated from European rabbits, hence share the same center of origin. The aim of the current study was to assess the genetic diversity and the origins of the domesticated rabbits in Kenya using mtDNA genetic marker.

## 2. Materials and Methods

### 2.1. Study Area and Experimental Design

Clearance for this research was granted by NACOSTI permit number NACOSTI/P/15/62961/8524. A purposive sampling strategy was adopted during the field surveys and collection of blood samples. Local and exotic rabbits were sampled from farmers practicing backyard small-scale rabbit farming in Kenya. Rabbits between 6 months and 5 years old, a male and a female whenever possible were sampled from each homestead. In some homesteads, only 1 rabbit was sampled due to genetic relatedness among the rabbits. A total of 111 rabbits were sampled from Kakamega, Vihiga, and Bungoma counties in the western region, Laikipia and Nyandarua counties in the central region, and Kitui, Machakos, and Makueni in the eastern region of the country.

### 2.2. Blood Sampling

The selected rabbit was restrained, and blood was drawn from the marginal vein of the ear. The needle was jabbed and blood drawn to a 75% filling of the 10 ml syringe. All the blood was applied on four spots on a labelled FTA classic card (Whatman Biosciences) for each rabbit and allowed to dry under a shade. The FTA cards were packaged in paper envelopes with silica gel desiccant and transported to the laboratory. The FTA cards were stored away from direct sunlight at room temperature until they were used for DNA extraction.

### 2.3. DNA Extraction, Amplification, and Sequencing

mtDNA was extracted using the Chelex protocol [17]. DNA concentration and quality were determined on a nanodrop. A 263-base pair region of mtDNA D-loop region, flanking the tRNA-Pro gene control region was amplified using CCACCATCAGCACCCAAAGCTG and TGGGCCCGGAGCGAGAAGAG [18] primers. PCR amplifications were carried out in 25 *μ*L reaction volume containing 2.5 *μ*L of 125 ng DNA, 5ul 5X Q5 buffer, 2 *μ*L 10 mM dNTPs, 1.25 *μ*L each 10 *μ*M forward (CCACCATCAGCACCCAAAGCTG) and reverse (TGGGCCCGGAGCGAGAAGAG) primers, 12.4 *μ*L PCR grade water, and 0.6 *μ*L (2 U/*μ*L) Q5High Fidelity DNA polymerase (New England Biolabs). Amplification conditions were set as follows: initial denaturation at 95°C for five minutes, 35 cycles of 95°C for 15 seconds, 60°C for one minute, and 72°C for two minutes and a final extension at 72°C for seven minutes on a PEQLAB thermocycler. The amplicons were purified using a DNA purification kit (ThermoFisher Scientific), and their concentrations were measured on a nanodrop. These concentrations were standardized to at least 50 ng/*μ*L and sent for sequencing at Macrogen, the Netherlands.

### 2.4. Data Analyses

Trimming sequences of the amplified fragment from Macrogen on CLC Main Workbench 7.9.1 (QIAGEN) resulted in 111 sequences. Subsequent comparisons and analyses were restricted to a 263-base pair region incorporating the hypervariable segment I (HVSI) from position 15,595 to 15,858. The contig sequences generated on CLC Main Workbench 7.9.1 were aligned using Clustal W in MEGA 6.0 against *Oryctolagus cuniculus* mitochondrion complete genome (accession number NC_001913.1) [19]. For comparison, mtDNA sequences of wild and domesticated rabbits from several locations were included in the data analysis [2].

Mitochondrial DNA haplotypes and their distribution in various regions, number of polymorphic sites, haplotype diversity, and nucleotide diversity were determined using DnaSP version 5.10.01 [20]. Genetic diversity of the rabbits in the sampled regions was determined by obtaining variation in the mtDNA. Intrapopulation diversity, number of polymorphic sites, number of haplotypes, haplotype diversity, and nucleotide diversity were first determined, and interpopulation diversity was visualized on a network profile. The origin of the rabbits was inferred phylogenetically on a Maximum-likelihood (ML) tree constructed using Mega 6.0 [19] using bootstrap of 1000 replications to provide for confidence in branching order. Pairwise F_ST_ was calculated to estimate genetic differentiation and hence the population genetic structure. Relationships among the haplotypes were further inferred on a haplotype network constructed using Population Analysis with Reticulate Trees (PopART) software [21]. Genetic differences among the sampled populations were tested using analysis of molecular variance (AMOVA) among and within the three populations [22]. The statistical significance of the AMOVA results was based on 1000 permutations.

## 3. Results

Alignment of the 263-bp fragment gave a total of 17 single nucleotide polymorphisms (SNPs) with 16 transitions and 1 transversion. These polymorphic sites generated eight unique haplotypes. Among the eight haplotypes observed in this study, haplotype seven was the most common, occurring in 55% individuals, followed by haplotype three in 36% of individuals. Haplotypes five and six occurred in one individual rabbit from Nyandarua and Machakos counties, respectively. Haplotype two occurred in the rabbit population from Vihiga County and among the pure breeds while haplotype eight was found among the rabbit population from Bungoma and Machakos counties. Haplotype distribution in the sampled regions is represented in Table 1.

All the haplotypes from the eight populations were polymorphic with the number of haplotypes ranging from two to four (Table 2). A wide range of nucleotide (0.00577–0.02041) and haplotype (0.166667–0.66667) diversities were revealed from the study populations. The least nucleotide diversity was from the rabbit population from Makueni (0.00577) while the highest nucleotide diversity (0.02041) was observed in rabbits from Vihiga. Haplotype diversity was equally least in rabbits from Makueni (0.16667) and highest in rabbits from Vihiga (0.66667). Rabbits from Machakos and Nyandarua counties had the highest number of haplotypes (4 in each of the population), whereas Makueni and Kitui had the least number of haplotypes (2 in each rabbit populations). Regional intrapopulation diversity (haplotype and nucleotide diversities) was highest in western and least in eastern, as shown in Table 3.

Population structure as revealed by pairwise FST estimates revealed that the rabbits sampled from the eastern and central regions had the least genetic distance (−0.01181) compared with rabbits from eastern and western regions being more genetically distant (0.09382).

### 3.1. Origin of the Rabbits

**Figure 1:**
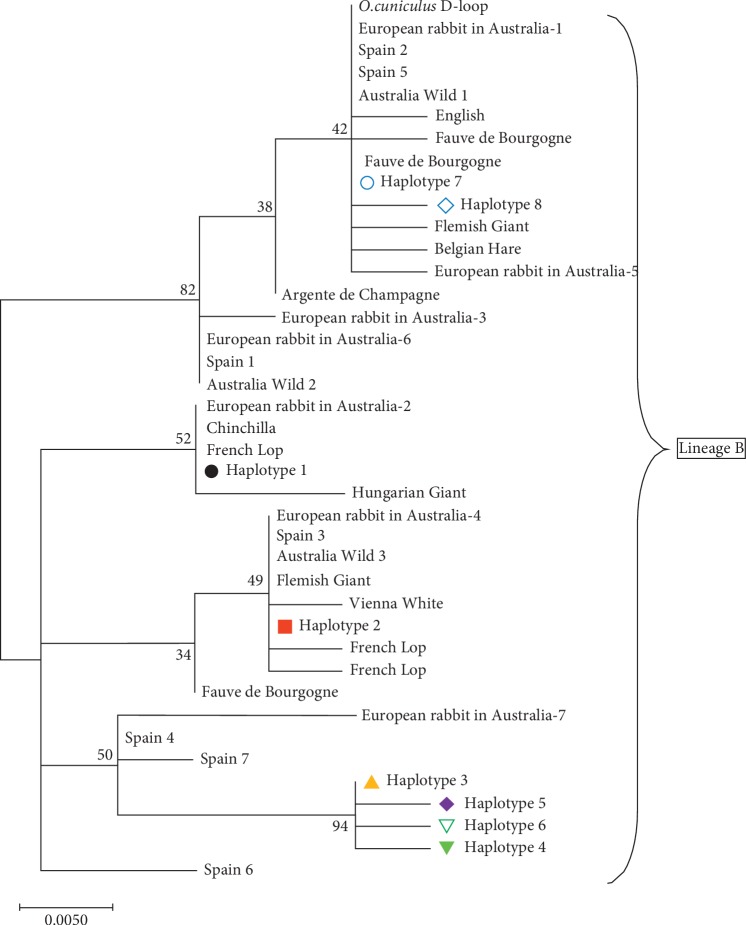
Unrooted Maximum-likelihood (ML) tree of the eight haplotypes identified in this study and the 32 reference sequences included for comparison. The numbers on the branches are percentages of bootstrap values with 1000 replications.

Inference of the origins on the unrooted Maximum-likelihood tree incorporating the eight haplotypes and the 32 sequences downloaded from GenBank is shown in Figure 1 below. One lineage was discerned, lineage B.

**Figure 2:**
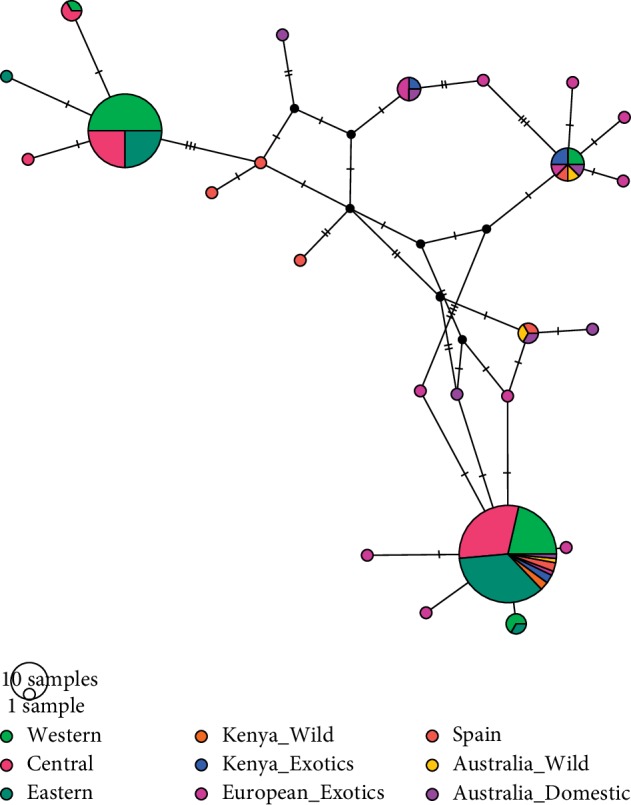
Haplotype network representing the association among the haplotypes based on the mtDNA D-loop region. Each circle represents a haplotype, with the size of the circle being proportional to the frequency of each haplotype. The sampling regions are color-coded as stated in the key. The threads between the haplotypes represent number of mutations.

Alignment of the eight haplotypes and published sequences of the European rabbit revealed a total of 24 unique haplotypes. The current study obtained five new haplotypes (H3, H4, H5, H6, and H8), whereas the other three haplotypes (1, 12, and 23) are identical to previously published haplotypes [2]. The relationships among the haplotypes and population structure were inferred on the haplotype network (Figure 2).

Analysis of molecular variance (AMOVA) among and within the populations revealed a significant genetic differentiation among and within the studied regions *p*=0.035 (<0.05), suggesting a significant genetic difference in the studied populations and the incorporated haplotypes.

## 4. Discussion

### 4.1. Population Genetic Diversity and Structure

The two indices for evaluation of mtDNA variation and genetic diversity of populations or breeds are haplotype diversity (Hd) and nucleotide diversity (Pi). Overall, the average haplotype (0.40702) and nucleotide (0.01494) diversities observed were low, indicating low genetic diversity of domesticated rabbits in Kenya. This could be attributed to breed exchange between the domesticated and exotic breeds by farmers. To confirm an expected effect of substantial structuring and low genetic diversity, it was observed that the number of rabbits that shared haplotype 3 was 40 whereas 61 individuals shared haplotype 7. Similar results were reported by Long et al. [2], where a low genetic diversity was observed in the three Chinese domestic breeds and introduced strains. In this study, 19 strains shared haplotype A1 and three strains shared haplotype A2. Intrabreed sequence analysis revealed 16 transitions and 1 transversion with no insertions or deletions. The transition type of mutation has been reported to be more common among very close relatives such as species within genus [23]. In this present study, transitions were the most frequent mutation, suggesting genetic closeness.

Other causes of low genetic diversity include founder effects during population expansion because of domestication and high selection pressure during commercial animal production which leads to an inherent decrease in strain variability [2]. Reduced strain variability directly results in reduced livestock genetic variability which equally applies to rabbits, as reported by this study.

Low genetic diversity is not good for livestock and even crops' improvement since loss in diversity could lead to loss of some lineages which could be harboring desirable traits. A possible mitigation strategy is the establishment of pedigree records followed by deliberate breeding strategies to further diversify desirable traits that are becoming extinct. This study has revealed rare haplotypes that can be conserved to be exploited in future breeding strategies. The haplotype network revealed distinct and heterogeneous population structuring in the rabbits sampled from western, central, and eastern regions in Kenya.

AMOVA analysis among and within the entire population revealed a clear structuring in the population, suggesting underlying genetic differences in the population. The population genetic structure was further revealed on the haplotype network with population structuring and a heterogeneous population in rabbits sampled from western, central, and eastern regions of Kenya. The genetic structuring observed here can be attributed to the controlled breeding and mating in the sampled rabbit populations. This is because these rabbits are mostly caged or isolated, and as a result, they undergo a high degree of nonrandom mating and social structuring. Moreover, populations that are isolated undergo high differentiation, as observed by Hewitt [11] and in this study. This clustering could be attributed to the presence of pure breeds among the farmers and the potential exchange of breeding stocks. Population genetic structure of domesticated rabbits is important in informing loss of genetic diversity that results from two processes: breed formation and domestication [2].

### 4.2. Origin and Evolution of Domesticated Rabbits in Kenya

Rabbit domestication has been attributed to a single origin in France [3]. Biju Duval et al. [13] reported two maternal lineages, A and B, in the domesticated rabbits. Lineage A mainly consists of wild rabbits belonging to subspecies *Oryctolagus cuniculus algirus* whereas lineage B is predominantly the subspecies *Oryctolagus cuniculus cuniculus* [2]. All domestic rabbits belong to the second lineage [15]. Phylogenetic analyses in this study revealed that the sampled rabbits belonged to lineage B.

Native rabbits from Middle Egypt belong to lineage A [12]. The study by [12] agrees with [11] who reported that the European wild rabbit originated in south Spain and North Africa.

In the current study, we inferred that domesticated rabbits in Kenya belong to lineage B and originated in Europe. Network analysis of the phylogenetic relationship of the eight haplotypes detected in this study and sequences retrieved from GenBank revealed associative patterns with the European rabbits, herein referred to as European exotics, such as rabbit in Spain and Argente de Champagne, whose origin is Western Europe [5]. The results obtained here confirm that Kenyan domesticated rabbits originated from European rabbits.

## Figures and Tables

**Table 1:** Haplotype distribution in the sampled rabbits from various counties.

Region/haplotype	Vihiga	Kakamega	Bungoma	Kitui	Machakos	Makueni	Nyandarua	Laikipia	Total
H2	2	0	0	0	0	0	0	0	**2**
H3	6	12	2	2	7	1	3	7	**40**
H4	0	1	0	0	0	0	1	1	**3**
H5	0	0	0	0	0	0	1	0	**1**
H6	0	0	0	0	1	0	0	0	**1**
H7	5	3	7	6	8	11	12	9	**61**
H8	0	0	2	0	1	0	0	0	**3**
Total	**3**	**3**	**2**	**2**	**3**	**2**	**3**	**3**	**111**

**Table 2:** Diversity indices and haplotype distributions in rabbits sampled from various counties.

Region	Sample size	Lineage	Number of polymorphic sites	Number of haplotypes	Haplotype diversity	Nucleotide diversity
Vihiga	13	B	11	3	0.66667	0.02041
Kakamega	16	B	10	3	0.42500	0.01173
Bungoma	11	B	10	3	0.58182	0.01259
Machakos	17	B	11	4	0.63971	0.01923
Makueni	12	B	9	2	0.16667	0.00577
Kitui	8	B	9	2	0.42857	0.01484
Nyandarua	17	B	11	4	0.49265	0.01618
Laikipia	17	B	10	3	0.58088	0.01878

**Table 3:** Intrapopulation diversity of the 111 rabbits grouped regionally.

Region	Number of sequences	Number of segregating (polymorphic) sites (S)	Number of haplotypes	Haplotype diversity	Nucleotide diversity
Eastern	37	11	4	0.48198	0.01528
Western	40	13	5	0.61923	0.01887
Central	34	11	4	0.54367	0.01751

## Data Availability

The data used to support the findings of this study are included within the article and were borrowed from other articles, appropriately cited in the bibliography.
